# Immune checkpoint inhibitor treatment – real-world use and outcomes in Sweden 2015–2023

**DOI:** 10.48101/ujms.v131.14245

**Published:** 2026-08-12

**Authors:** Kristoffer Illergard, Pablo Soto, Johannes Arpegård, Aglaia Schiza, Simon Pahnke

**Affiliations:** a Division of Research, Informatics & Visualization, Reveal AB, Stockholm, Sweden; b Department of Oncology, Uppsala University Hospital, Uppsala, Sweden; c Department of Immunology, Genetics and Pathology, Uppsala University, Uppsala, Sweden

**Keywords:** Immune checkpoint inhibitors, real-world outcomes, cancer treatment

## Abstract

**Background:**

The introduction of immune checkpoint inhibitor (ICI) treatment has significantly improved the treatment of several cancer diagnoses, such as non-small cell lung cancer, renal and urothelial carcinoma, melanoma. However, there is a lack of real-world data on ICI treatment and outcomes from the Swedish healthcare setting. The cost of treatment is also significant, and securing equal access to emerging drugs for all patients is challenging in an era of rapidly evolving treatment recommendations.

**Methods:**

We conducted a population-based observational study of incident cases among ICI-treated patients, using a novel electronic healthcare record source for systemic cancer drugs linked to regional and national register data. Overall survival (OS) was calculated from start of the first ICI-treatment, using the Kaplan-Meier method. The aim was to examine treatment with ICI drugs during 2015–2023 in 10 Swedish regions (total population 4.3 million).

**Results:**

Median real-world OS was 2.0 years (95% confidence interval: 1.8–2.2 years) from start of ICI-treatment for patients with known palliative treatment intent (*n* = 1,941, lung 2.2 years, melanoma 5.0 years, renal 2.3 years, urothelial 1.1 years, head/neck 1.4 years).

In 2023, 6.7% of all patients receiving systemic antineoplastic treatment for cancer were treated with ICI (74 per 100,000 inhabitants). The timing of the adoption of treatment with ICI drugs and the current patterns of use differed between Swedish regions.

**Conclusion:**

This study provides the first Swedish real-world OS estimates after ICI-treatment across major diagnoses, documents the rapidly increasing ICI use, and describes regional variation in treatment patterns.

## Introduction

Treatment with immune checkpoint inhibitors (ICI) has shown impressive improvements in overall survival (OS) for patients with advanced stage cancer in large randomized clinical trials (RCT) (1–4).

The concordance between RCT and real-world assessed outcomes tends to be relatively high (5, 6); however, real-world data for ICI treatment are only starting to emerge, and so far only for some diagnoses (7, 8). To date, no data on real-world outcomes of ICI treatment have been published from the Swedish healthcare setting.

The introduction of novel cancer treatments can impose substantial economic pressures on healthcare systems (9). In Sweden, a taxpayer funded universal health coverage provides access to drugs approved by the European Medical Agency only after a health-economic analysis and reimbursement decision by The Dental and Pharmaceutical Benefits Agency (Tandvårds- och läkemedelsförmånsverket [TLV]). Scientific group guidelines, as well as regional and local directives, may all ultimately influence how new drugs are then used (9).

Geographical equity, and the timely integration of novel treatments, have been emphasized as crucial for improving health outcomes in several countries, including Sweden (10, 11). Inefficient allocation of healthcare resources could raise the economic burden, and socioeconomic factors have been found to influence disparities in healthcare access and utilization (12–14).

Our study explores the adoption of ICI treatment for cancer across 10 Swedish regions. It provides real-world estimates of OS for patients receiving ICI therapy, and analyses potential regional variations in treatment use.

## Materials and methods

### Design

We conducted a population-wide, register-based cohort study across 10 of Sweden’s 21 regions, encompassing a total population of approximately 4.3 million. All Swedish regions where relevant data were accessible at the time of the study were included.

### Inclusion criteria and data sources

We identified all patients with a cancer diagnosis (International Classification of Diseases [ICD] codes ‘C00–C99’) in the Swedish National Patient Register (NPR) (15) or those having been administered or prescribed a cancer drug (Anatomical Therapeutic Chemical classification [ATC] ‘L01 Antineoplastic drugs’) according to the electronic healthcare records of 10 regions: Östergötland, Uppsala, Skåne, Västerbotten, Dalarna, Blekinge, Västmanland, Halland, Kronoberg and Norrbotten or the Swedish National Prescribed Drug Register (SPDR) (16)), between January 2015 and December 2023.

Mortality data, including dates of death, were retrieved from the Cause of Death Register (COD) (17), and cancer diagnoses were verified using the Swedish National Cancer Register (18, 19).

Treatment intent (curative: adjuvant 72%, neo-adjuvant 21%, other 7% or palliative) for patients receiving ICIs was obtained from the Cancer Drugs Register (CDR) (20); data were available for 2,473 patients.

The linkage of register data at individual level was performed by the Swedish National Board of Health and Welfare using patients’ unique Personal Identity Numbers (PINs); the data were pseudonymized before analyses.

### Datasets

Two datasets, as illustrated in Supplementary Figure 1, were created as follows:

1. **Target dataset**: Comprised patients who received an ICI between 2015 and 2023.
2. **Background dataset**: Included all patients from the 10 regions who had received either a cancer drug (ATC code L01) or had a registered cancer diagnosis during the study period.
  Exclusion criteria were then applied as follows:
  - Patients with a cancer diagnosis but no recorded treatment with systemic antineoplastic drugs (e.g. patients not receiving systemic treatment or receiving only anti-hormonal therapies).
  - Patients treated with systemic antineoplastic drugs without a registered cancer diagnosis.

### Statistical analysis

We calculated the number of incident cases of patients treated with any ICI-drug during the study period – ‘treated patients’ – according to the stratification groups: region, cancer diagnosis, and year of treatment.

To facilitate comparisons across regions with varying population sizes, standardization was conducted using two methods: (1) calculating the number of ICI-treated patients per 100,000 inhabitants in each region, and (2) determining the proportion of patients receiving systemic cancer treatment who were treated with ICIs.

For the survival analyses that were based on treatment intent, data on ICI treatment intent (palliative/curative) were collected from the CDR where available (2,473 of 8,641 ICI-treated patients).

Visualization and statistical analyses, such as Pearson’s Chi-squared test, were performed in R (21). OS from start of the first ICI-treatment was calculated using the Kaplan-Meier method, as implemented in the *survival* package in R (22).

## Results

### Patient characteristics

Between January 2015 and December 2023, a total of 8,641 patients were treated with ICIs, while 129,724 patients received some form of systemic cancer therapy. The overall number of patients treated with ICI, the corresponding number treated per 100,000 inhabitants, and patient characteristics stratified by cancer diagnosis group, are detailed in Table 1.

**Table 1:** Distribution of patients by diagnosis groups for study time period, 2015–2023.

Category	Cancer diagnosis group	ICD-10 code	Patients treated with ICI, *n*	Patients with systemic cancer treatment, *n*	ICI-treated per 100 patients treated for cancer	ICI-treated per 100,000 inhabitants	Mean age	Male ratio, %
Totals			8,641	129,724	6.7	199.6	68.1	53.9
Diagnoses with currently approved indication	Lung	C34	3,462	9,919	34.9	80.0	70.9	46.5
	Melanoma	C43	1,815	2,494	72.8	41.9	65.9	58.8
	Renal	C64	733	1,417	51.7	16.9	67.1	71.8
	Urothelial	C65–C68	590	3,023	19.5	13.6	71.9	68.1
	Breast	C50	338	30,413	1.1	7.8	55.6	0.3
	Head/Neck	C1–C14, C30, C32	247	1,351	18.3	5.7	64.5	72.1
	Skin, non-melanoma	C44	201	2,775	7.2	4.6	74.0	66.7
	Colorectal	C18–C20	172	9,958	1.7	4.0	67.8	43.6
	Mesothelioma	C45	121	369	32.8	2.8	73.2	85.1
	Ventricular	C16	120	1,565	7.7	2.8	66.5	72.5
	Esophagus	C15	113	1,181	9.6	2.6	68.1	69.0
	Liver	C22	101	800	12.6	2.3	65.5	75.2
	Hodgkin’s lymphoma	C81	68	824	8.3	1.6	54.3	69.1
	Cervical	C53	47	944	5.0	1.1	53.8	0.0
	Endometrial	C54	47	1,749	2.7	1.1	66.5	0.0
	Totals, approved indications		8,175	68,782	11.9	188.8	68.3	53.6
Diagnoses without currently approved indications	Totals		466	60,942	0.8	10.8	64.1	59.4

ICI: immune checkpoint inhibitor.

In 2023, 6.7% of all patients receiving systemic cancer therapy were treated with an ICI (3,191 of 47,886 patients, Supplementary Table 1). In diagnosis groups with a currently approved indication for ICI treatment, 11.4% were treated with an ICI (3,018 of 26,584 patients).

Supplementary Figure 2 shows the absolute number of ICI-treated patients per region during the study period.

### Real-world OS after initiation of ICI treatment

For all patients treated with an ICI, median OS was 4.7 years from initiation of ICI treatment (1,713 days, 95% confidence interval [CI]: 1,559–1,937), 1-year OS 74%, 2-year OS 61%, and 5-year OS 49%, Supplementary Table 1).

**Figure 1:**
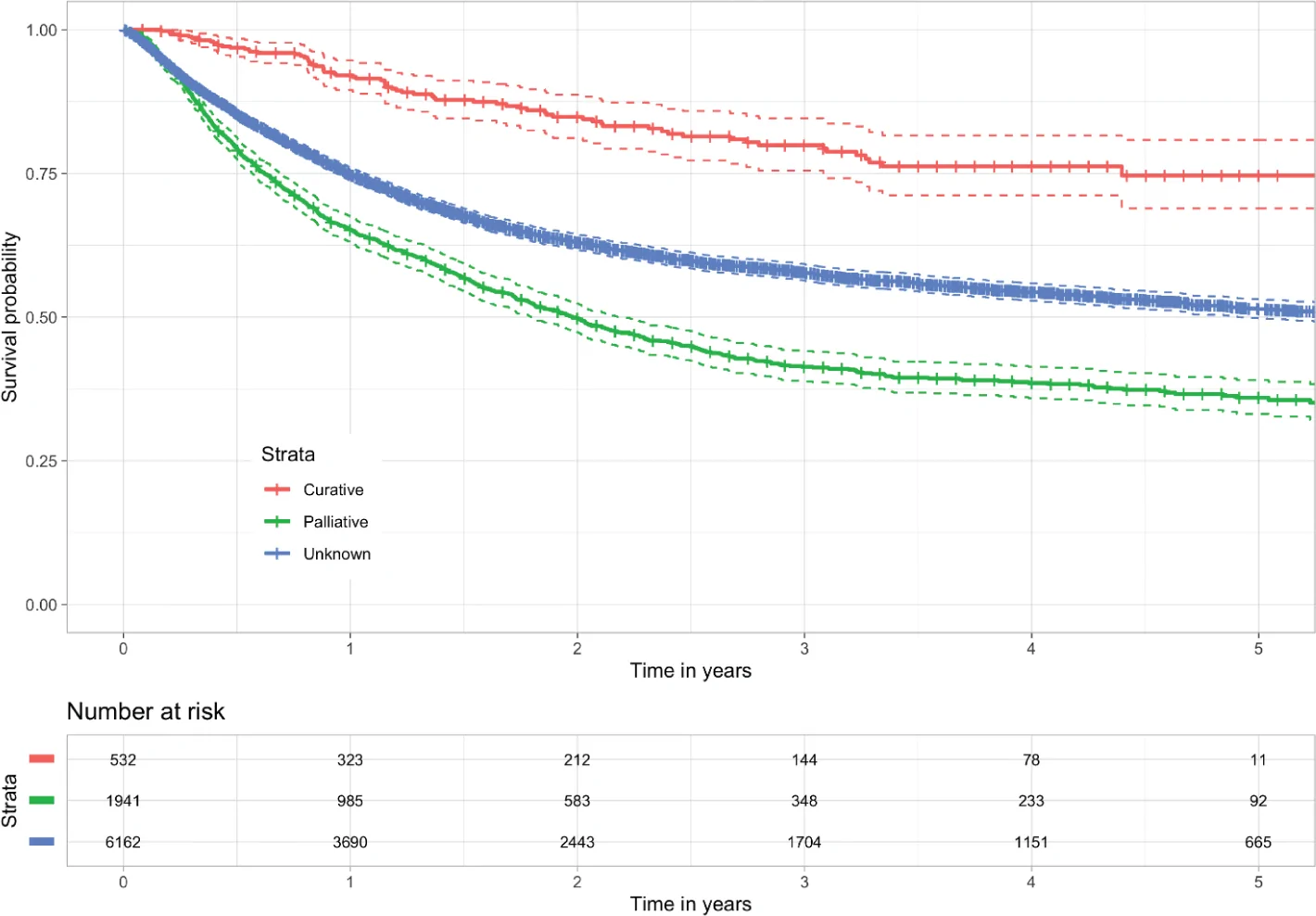
Overall survival, all diagnoses, by treatment intention.

**Figure 2:**
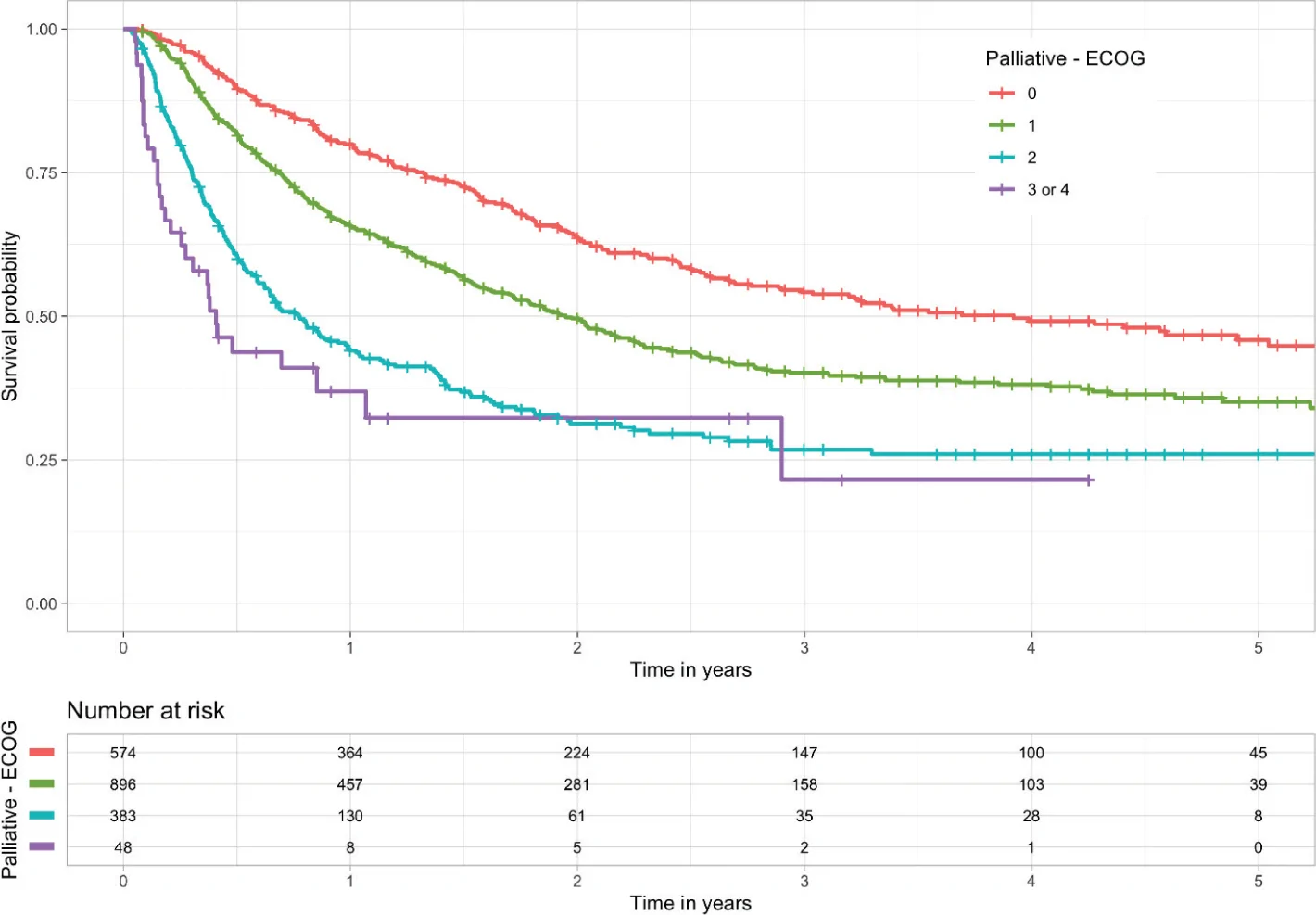
Overall survival, palliative ICI treatment, by ECOG status. ICI: immune checkpoint inhibitor.

For patients with known palliative treatment intention (*n* = 1,941), median OS was 2.0 years (723 days; 95% CI: 656–795), 1-year OS 65%, 2-year OS 50%, and 5-year OS 36% (Figure 1, Supplementary Table 1). Among patients receiving palliative ICI treatment, poorer Eastern Cooperative Oncology Group (ECOG) performance status at treatment initiation was associated with shorter observed OS. Median OS decreased progressively across ECOG groups, from 3.9 years (1,432 days; 95% CI: 1,059–not estimable [NE]) in patients with ECOG 0, to 1.9 years (711 days; 95% CI: 628–814) for ECOG 1, 0.8 years (283 days; 95% CI: 227–361) for ECOG 2, and 0.4 years (149 days; 95% CI: 100–NE) for ECOG 3–4 (Figure 2 and Supplementary Table 1). Similar patterns were observed in the two largest diagnosis groups — melanoma and lung cancer. The other diagnosis groups were too small to analyze by ECOG status subgroups

**Figure 3:**
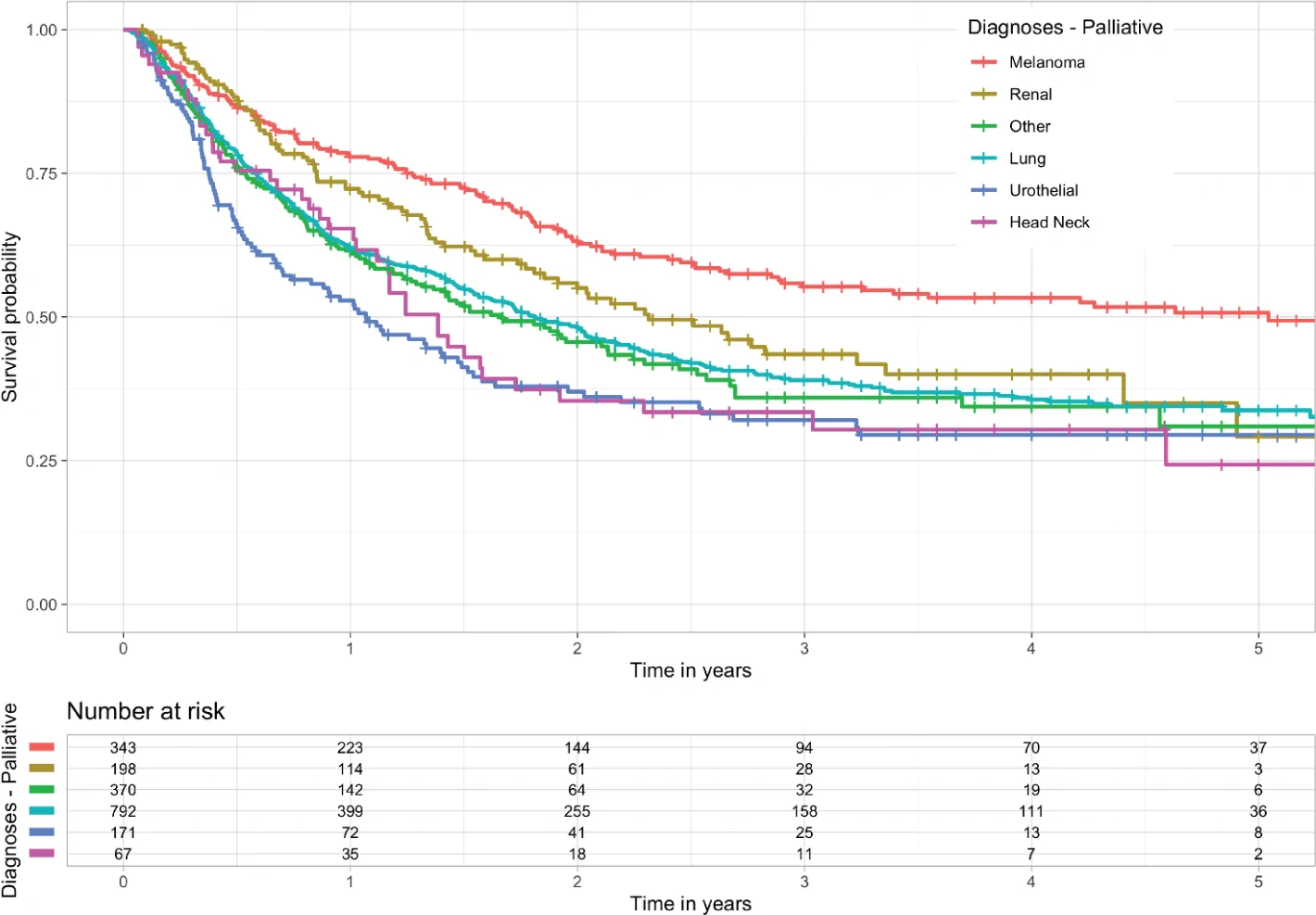
Overall survival, palliative ICI treatment, by diagnosis group. ICI: immune checkpoint inhibitor.

Specifically, 48 patients with a performance status of 3 or 4 were recorded as receiving palliative ICI treatment, with a median OS of 4.9 months (149 days, CI: 100, NE). Eight and five of these patients were alive and had a follow-up beyond 1 and 2 years after start of ICI treatment, respectively. Median OS for patients receiving palliative ICI treatment varied significantly by cancer diagnosis group. The highest OS was observed for patients with melanoma [5.0 years (1,842 days; 95% CI: 1,091, NE)], followed by renal cell carcinoma (RCC) [2.3 years (845 days; 95% CI: 677–1,609)], lung cancer [1.8 years (390 days; 95% CI: 257–554)], head/neck cancer [1.4 years (506 days; 95% CI: 408–837)], and urothelial cancer [1.1 years (663 days; 95% CI: 571–774)] (Figure 3, Supplementary Table 1). Five-year OS rates ranged from 51% in melanoma patients to 24% in those with head/neck cancer.

**Figure 4:**
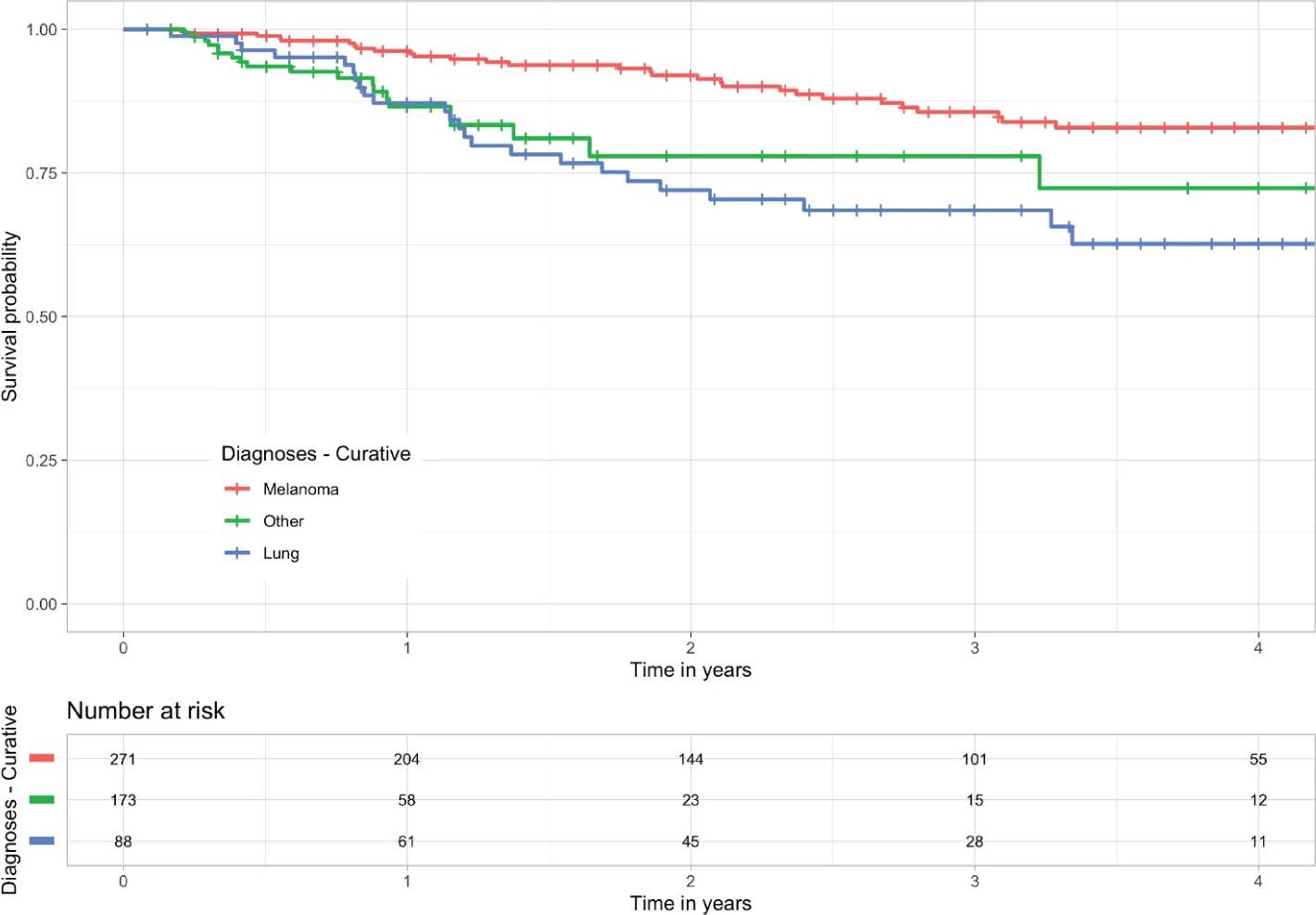
Overall survival, curative ICI treatment, by diagnosis group. ICI: immune checkpoint inhibitor.

For patients receiving curative ICI treatment (*n* = 532), median OS was not reached, with 5-year survival rates for all patients, and those with melanoma or lung cancer of 75, 80, and 63%, respectively. (Figures 1 and 4, Supplementary Figures 5 and 6, Supplementary Table 1).

### Adoption rates and current use of ICI treatment

**Figure 5:**
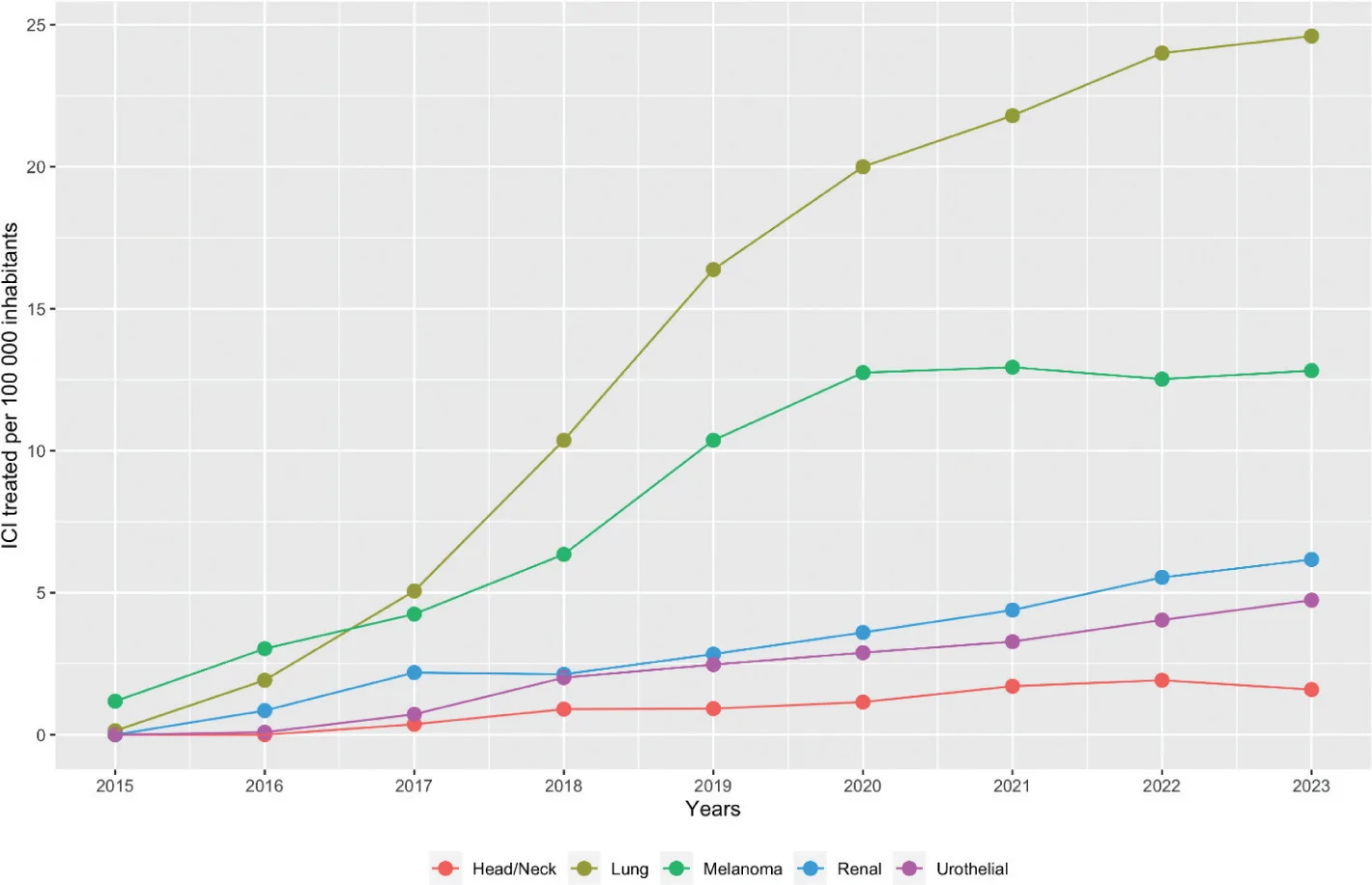
Number of patients treated with ICI per 100,000 inhabitants, by year and diagnosis group, top 5 diagnoses. ICI: immune checkpoint inhibitor.

**Figure 6:**
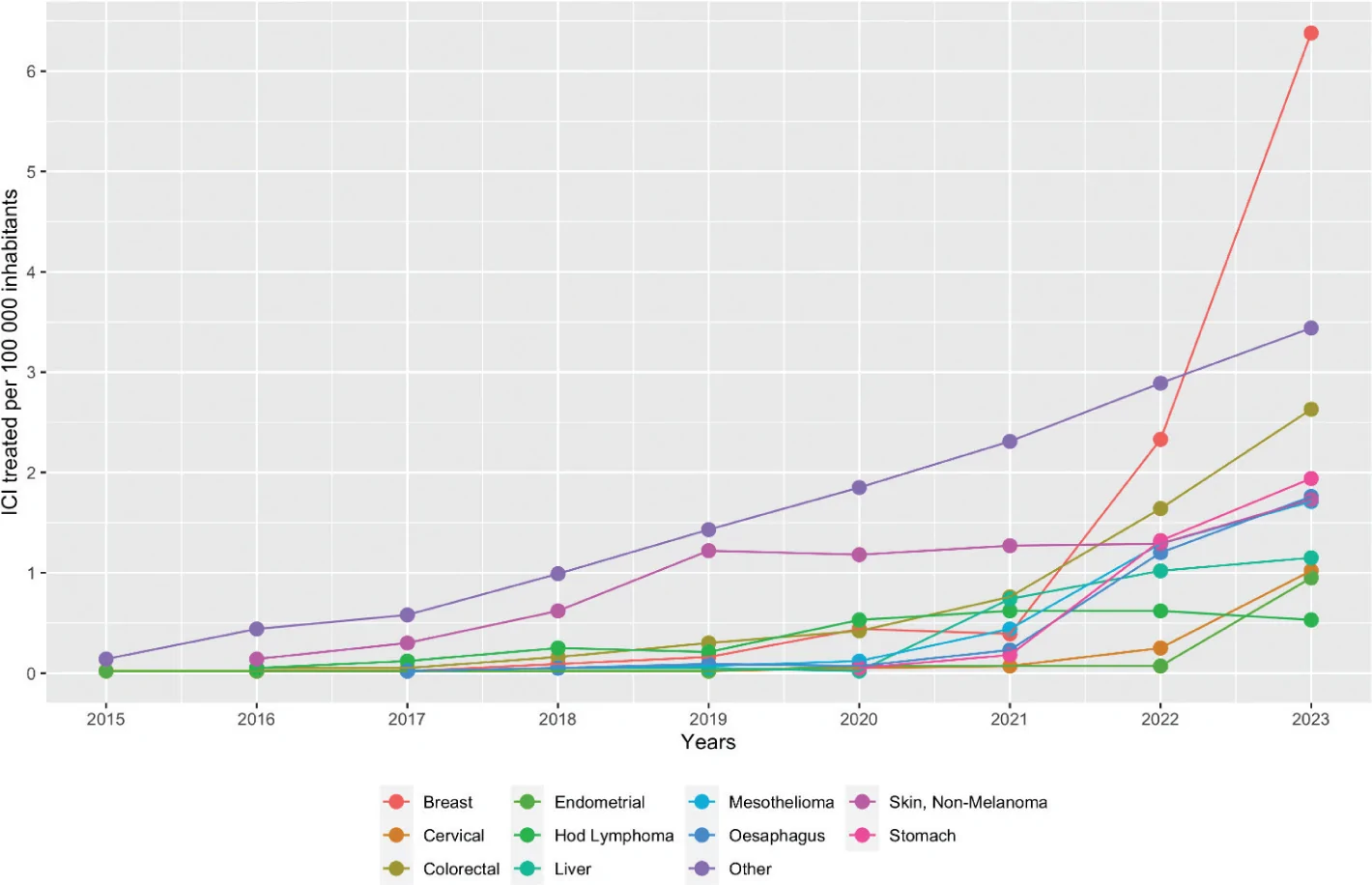
Number of patients treated with ICI per 100,000 inhabitants, by year and diagnosis group, non-top 5 diagnoses. ICI: immune checkpoint inhibitor.

The use of ICIs increased rapidly between 2015 and 2023 (Figure 5—Top 5 most common diagnoses, and Figure 6—non-Top 5 diagnoses). A nearly linear increase in ICI treatment was observed for four of the five most commonly treated cancer diagnoses, although the trend appears to plateau for melanoma.

**Figure 7:**
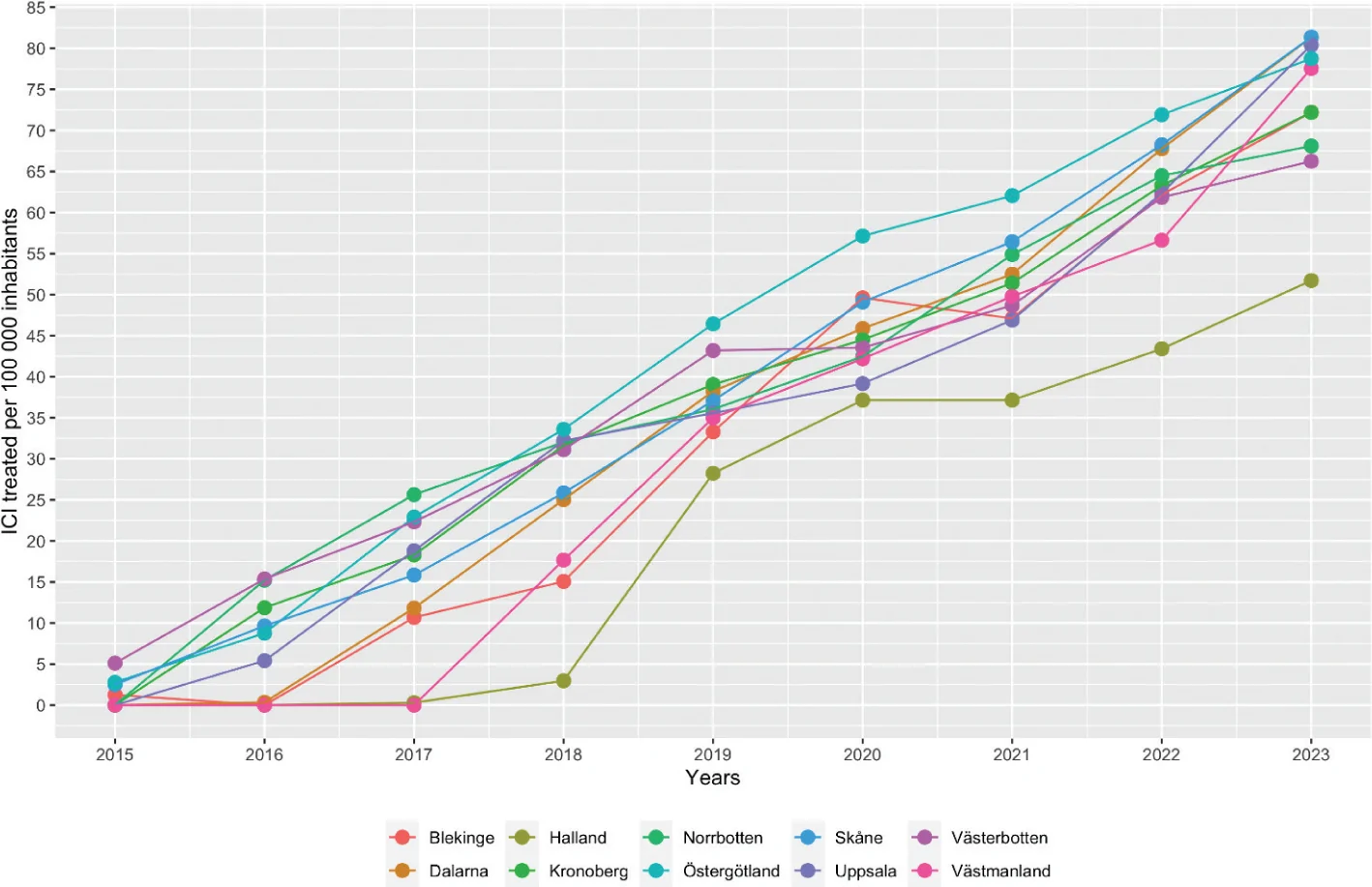
Number of patients treated with ICI per 100,000 inhabitants, per region. ICI: immune checkpoint inhibitor.

In 2023, ICI usage rates varied significantly across regions. The proportion of patients receiving ICI treatment ranged from 81 per 100,000 inhabitants in Region Skåne to 52 in Region Halland (Chi2 test *P* < 0.0001) (Figure 7).

There was also a pronounced variation in the proportion of patients being treated with ICI across cancer diagnosis groups. In 2023, the patients most likely to receive ICI treatment were those with melanoma (65.3%), renal (53.6%), lung (43.1%), urothelial (29.2%), or head/neck cancer (23.1%) (Supplementary Figure 7: Top 5 diagnoses and Supplementary Figure 8: non-Top 5 diagnoses, and Supplementary Table 2).

Among ICI drugs, Pembrolizumab (3.4% of all patients treated for cancer) and Nivolumab (2.1%) were the most commonly used in 2023 (Supplementary Figures 9 and 10).

## Discussion

### Real-world outcomes of ICI treatment

ICIs have significantly improved prognosis for many cancer patients. Evaluating how real-world OS data compare to results from pivotal clinical trials is crucial for understanding the effectiveness of these therapies beyond the controlled trial environment. Swedish national healthcare and quality registers have been extensively used for studying real-world outcomes of cancer treatments (23–26). However, except for prescribed oral drugs, these registers depend on the manual registration of administered treatments and typically lack treatment information data beyond first line.

Our study is the first to collect systemic cancer treatment data directly from Swedish regional electronic cancer treatment databases, enabling us to capture all systemic cancer treatments regardless of line of treatment. We believe this study could serve as a proof-of-principle that this novel data source can be a valuable tool for real-world cancer treatment studies, especially when combined with other national registers.

Our data does not allow us to perform proper statistical comparisons to pivotal clinical trials, adjusting for possible confounders for each diagnosis and study, but some broad comparisons can be made for context.

### Non–small cell lung cancer

In large randomized non–small cell lung cancer (NSCLC) trials, the 1-year OS rates for first and second-line ICI treatments in the palliative setting ranged from 42 to 70%, with median OS ranging from 12.2 to 30 months (27, 28). Our findings seem to fall in the same range, with a 1-year OS rate of 62% and a median OS of 21.8 months, although the patients in our analysis could have received ICI treatment as any line of therapy.

## Melanoma

Randomized trials report 1-year OS rates of 73% and 2-year OS rates of 43–55% for single PD-1 inhibitors (1, 2), while combination ICI therapy shows slightly higher rates, with a 1-year OS of 83% and a 2-year OS of 64% (29). These results align with our study, which found a 1-year OS of 78% and a 2-year OS of 63% for palliative ICI treatment, although treatment with single PD-1 inhibitor or as combination ICI is not analyzed separately.

### Renal cell cancer

Patients with RCC, had a median OS of 27.8 months and a 2-year OS rate of 55% for patients receiving palliative ICI treatment. These results align with clinical trial results of second-line nivolumab treatment (median OS 25 months, 2-year OS 55%) (30), but seem lower than that reported for first-line combination therapies, such as ipilimumab and nivolumab (median OS 47 months, 5-year OS 43%) or nivolumab with cabozantinib (1-year OS 85.7%) (31, 32). However, it should be noted that in our study population, we do not distinguish between patients that received ICI alone or in combination with other medications, nor do we have data on the treatment line when they received ICI. Patients with RCC had a median OS of 27.8 months and a 2-year OS rate of 55% following palliative ICI treatment. Reported outcomes in pivotal RCC trials have varied substantially depending on treatment line and regimen. The survival outcomes observed in our cohort fall within the range reported in these studies, although direct comparisons are limited by the lack of data on treatment line and combination regimens in our dataset

### Urothelial cancer

Our study found a median OS of 9.6 months and a 1-year OS rate of 53% for patients with urothelial cancer, regardless of the treatment line they received, similar to what has been reported for 2^nd^ line treatment with Pembrolizumab treatment for advanced urothelial carcinoma (median OS 10.3 months, 1-year OS 44%) (33).

### Head/Neck cancer

For head and neck cancers, median OS was 16.6 months, with 1-year and 2-year OS rates of 65 and 35%, respectively. Reported median OS in pivotal trials ranged from 7.5 months for nivolumab treatment to approximately 11.5–13 months for pembrolizumab-based first-line treatment approaches (34). However, direct comparisons should be interpreted cautiously due to differences in patient selection and treatment setting.

### OS of very frail patients

Our data included 48 patients with a performance status (ECOG) of ≥ 3 at treatment initiation, a group typically excluded from clinical trials. These patients had a median OS of just 149 days.

While treatment in this frail cohort poses increased risks, and might cause more harm than good (35), these findings could indicate that some patients may still benefit from treatment. Eight patients with ECOG ≥ 3 survived beyond 1 year, and five beyond 2 years. This indicates that some carefully selected frail patients might derive benefit from ICI treatment, underscoring the importance of individualized and thorough discussions before initiating therapy.

### Rapidly increasing use of ICI treatment

Between 2015 and 2023, ICI usage increased rapidly, reaching 6.7% of all treated cancer patients, or 74 per 100,000 inhabitants, in 2023. With new indications and novel targets for ICIs, further increases in the number of eligible patients are anticipated (36).

The cost of ICI treatment represents a growing challenge. In 2023, reimbursement for ICI drugs in Sweden reached approximately 2.9 billion Swedish Krona (SEK) (United States dollars [USD] 290 million), although this excludes confidentially negotiated price reductions (37).

### Regional differences in use of ICI drugs

Our study identifies regional differences in ICI use across Sweden. These could reflect natural variations due to population demographics, disease prevalence, or diagnostic stages, as well as differences in patterns of use between local healthcare organizations. As an example, the region with the lowest number of ICI-treated patients per 100,000 inhabitants (Halland) does not treat advanced stage melanoma patients. However, in some instances, these disparities may also indicate inequitable access to novel treatments.

Regional differences in the adoption of new cancer treatments in Sweden have previously been observed for other cancer therapies, such as bevacizumab, partly attributed to differences in treatment traditions [9]. Differences between countries are even more pronounced, with lower adoption of new cancer drugs in lower-income geographical regions (38).

While our study was not designed to explore in detail the underlying causes of regional variation in use, we believe the findings highlight a need for further investigations into potential barriers to equitable access to novel cancer treatments.

### Strengths and limitations

The key strength of this study lies in its design as a population-wide cohort, using a novel database with automated collection of cancer treatment data directly from regional electronic cancer treatment databases. This enabled the identification of all ICI-treated patients (8,641) across 10 Swedish regions of varying population sizes. This approach ensures a broad, heterogeneous patient population, enhancing the generalizability of the findings. Additionally, the use of high-coverage national registers facilitates high data quality through linkage at individual level using patients’ unique Swedish PINs.

However, the study shares several limitations common to register-based research. Although the national registers used are generally of high quality and have validated coverage, the CDR has lower and highly variable regional coverage, affecting the completeness of our data. Treatment intent (from the CDR) was only available for 29% of all patients receiving ICI-treatment, and this missing data are likely not random which can lead to biased results. Designation of treatment intent in the CDR (curative vs. palliative) is also not clearly defined, and is left to the treating physician’s subjective judgment.

Additionally, our dataset does not allow for analysis of survival outcomes by tumor stage, or line of treatment, limiting the ability to compare OS results with those from clinical trials. The observational design of the study also limits causal interpretation of comparisons across diagnosis groups and treatment settings.

## Conclusions

During the first nine years of ICI treatment in Sweden, the year-by-year increase in ICI-treated patients has been dramatic, with 6.7% of all cancer patients being treated with systemic anti-cancer drugs in 2023.

Real-world OS following ICI treatment is broadly similar to that seen in pivotal clinical trials for diagnoses where ICI treatments are currently most common (lung, melanoma, renal, urothelial, and head/neck).

However, regional disparities in ICI adoption and usage underscore the need for targeted studies and efforts to ensure equity and optimal resource allocation within Sweden’s healthcare system. Future research should explore the underlying drivers of these variations in use, and evaluate strategies to address them, ensuring that all patients can benefit from advancements in cancer immunotherapy.

## Data Availability

Materials and data supporting the findings of this study are available from the corresponding author upon request, but release may be confined to limitations set by ethical authorities.
